# Young smoker “ABCD” vascular assessment: a four-step ultrasound examination for detecting peripheral, extra and intra-cranial early arterial damage

**DOI:** 10.1186/s12872-016-0318-z

**Published:** 2016-07-08

**Authors:** Chiara Mozzini, Alder Casadei, Giuseppe Roscia, Luciano Cominacini

**Affiliations:** Department of Medicine, Section of Internal Medicine, University of Verona, Piazzale L.A. Scuro, 10-37134 Verona, Italy; Ultrasound Association of South-Tyrol, Bolzano Health District, Piazza W.A. Loew-Cadonna, 12-39100 Bolzano, Italy; Department of Internal Medicine, Bolzano Central Hospital, via L. Bohler, 5-39100 Bolzano, Italy

**Keywords:** Smoking, Ankle-brachial index, Breath holding index, Carotid intima-media thickness, Abdominal aorta diameter, Early arterial damage

## Abstract

**Background:**

Cigarette smoking is known as a major risk factor in the pathogenic mechanisms of stroke, coronary and peripheral artery disease (CAD and PAD), even in young subjects. The aim of this study is the creation of a four-step ultrasound examination to evaluate and monitor the peripheral, the extra and the intra-cranial assessment of the arterial early damage in smokers. The evaluations of **A**, the *A*nkle-brachial index, *A*BI, **B**, the *B*reath holding index, *B*HI, **C**, the *C*arotid intima media thickness, *C*IMT, and **D**, the *D*iameter of the abdominal aorta represent the “**ABCD”** assessment.

**Methods:**

Thirty-eight healthy smokers and 43 controls underwent **A**, calculated for each leg. **B** was calculated after determination of subjects’ flow velocity of middle cerebral artery (MCA) by trans-cranial colour Doppler (TCCD) before and after 30 s of apnoea at baseline and just after smoking a cigarette, to simulate the chronic and acute effects of smoking. Finally, **C** and **D** evaluation were assessed using a high-resolution B-mode ultrasound.

**Results:**

Smokers presented higher values of CIMT (mean and maximal), and lower BHI both at baseline and just after smoking (*p* < 0.01), though in the normal range. No significant differences were found for A and D between smokers and non- smokers.

**Conclusions:**

Our results underline the importance of the assessment of *B* and *C*, that, though in the normal range, present significant differences between smokers and non-smokers. These data could drive the screening between smokers in age-related manner. Moreover, the “**ABCD”** examination could represent a valid method to detect and then monitor smokers’ vascular damage. Although it is far to be considered a screening and routine tool, it should be contemplated in a wider context of possible not-invasive practical screening and follow-up modalities. This would be designed to implement preventive strategies and tools aimed at discouraging tobacco addiction and monitoring cardiovascular risk patients.

## Background

Cigarette smoking is known as a major risk factor in the pathogenic mechanisms of stroke, coronary and peripheral artery disease (CAD and PAD), even in young subjects [1–3].

PAD can be assessed with not invasive tests and the ankle-brachial index (ABI) is the most widely used measure to detect the presence of PAD [4]. Originally described by Winsor [5], nowadays the ABI is an important indicator of atherosclerosis and a prognostic marker, even in absence of symptoms [6]. An ABI of less than 0.90 is the threshold to define PAD, using the Doppler modality [4].

Several studies have confirmed a dose and time-dependent relationship between carotid intima media thickness (CIMT) and smoking in the context of subclinical atherosclerotic damage [7]. In particular, our group [8] has recently demonstrated higher CIMT values in healthy young smokers than in age-sex matched non-smokers. CIMT, assessed by B-mode ultrasonography is a sensitive marker of atherosclerosis, also in the early stages, being an independent predictor of stroke and cardiovascular events [9].

Regarding the effects of smoking on the impairment of cerebral circulation, it is known that acute smoking reduces the cerebral vasodilator response to hypercapnia [10, 11], but the effects of chronic smoking are not well established. Cerebral vasomotor reactivity can be studied by measuring changes in flow velocity in response to vasodilatatory stimuli as such as CO_2_ inhalation, breath holding or acetazolamide administration [12, 13]. The breath-holding index (BHI) is considered the most practical, inexpensive, best-tolerated and easy to perform alternative to the previous methods [13].

Moreover, if a significant carotid stenosis or occlusion is considered as one of the most important factors affecting cerebral vasomotor reactivity, very recently an association between altered reactivity and CAD has been established, even in the absence of significant carotid stenosis [14].

Finally, it is well known for many years that smoking has also been emphasized as an independent risk factor for abdominal aortic aneurysm (AAA) [15], but precise worldwide-accepted recommendations for screening are still lacking [16].

This work intends to better define the vascular damage of smoking in young healthy people, creating a relatively easy ultrasound examination system, in order to provide evidence of the major impact of smoking on vascular assessment and to underline the importance of preventive strategies in the young population.

The same system (ABCD) could be the basis for future screening and follow up modalities also for older smokers and for the “vascular “risk patient.

## Methods

Ethics statement: the study was conducted in accordance with the ethical standards laid down in the Helsinki Declaration of 1975 and its late amendments. Healthy volunteers were recruited among the students of Verona University School of Medicine and among the Internal Medicine certification board students of the same institution. All participants provided written consent prior to commencing the study and the local ethical committee approved the study.

The healthy volunteers were recruited according to the following criteria: male and female; aged 23–31; normal BMI; no use of antioxidant supplementation or anti-inflammatory medication during or for six months prior to commencing the study; no diagnosed cerebral or cardio or peripheral vascular disease, hypertension, hypercholesterolemia or diabetes mellitus or endocrine disorders.

Non-smokers included those who had never smoked or those who had not smoked for at least 3 years.

Smokers were described as individuals who smoked at least 3 cigarettes per day with a smoking duration period ≥ 6 months.

Studies were conducted in a quiet, temperature-controlled laboratory (25 °C), in the morning. Subjects were asked to retrain from caffeine and alcohol-containing a beverages for at least 8 h before the examination. The examinations were conducted at baseline for the non-smokers and both at the baseline and within maximum of 10 min after smoking a cigarette for the smokers (based on previous data about the peak of blood nicotine concentrations in the systemic circulation after smoking [17]).

Subjects were tested as “with regular sinus rhythm clinically” (no history of arrhythmia or any cardiovascular disease was reported).

Ultrasound examinations were performed by the same examiner (CM) who was unaware of the subjects’ smoking habits before performing the A, baseline B, C and D.

The following steps constitute the ABCD examination.

### *A*: the ABI assessment

According to a recent review statement about its measurement [4], the ABI was determined from blood pressure measurement in both upper limbs and ankles, with the subject in supine position. Systolic blood pressure from the brachial artery in the antecubital fossa was measured using a blood pressure cuff and high-resolution B-mode ultrasound (HC Envisor, Philips) equipped with continuous –wave Doppler 5 MHz probe. In the lower limbs, systolic blood pressure from the posterior tibial or dorsalis pedis arteries was measured with Doppler detection by placing a blood pressure cuff at the ankle. ABI was calculated as the ratio of the higher systolic blood pressure measured at the ankle to the systolic blood pressure measured at the brachial artery. The interpretation of the ABI was made according to the current classification and interpretation recommendations [4].

### *B*: the BHI assessment

The subjects were examined while supine, with their head hyperextended and turned away from the side being scanned. The trans-temporal approach was used to detect continuous measurements of flow signals from the M1 segment of the middle cerebral artery (MCA), 45–65 mm depth [18] Pulsed-wave TCCD (2 MHz probe, Envisor, Philips) was used.

Cerebrovascular reactivity to hypercapnia was evaluated by means of the BHI [13]. The BHI has been calculated according to the following formula: BHI% = [(MFV *apnea—*MFV *rest)/*MFV *rest* x30] x 100*.*

The breath holding indices from the two sides were averaged within one subject.

Mean flow velocity (MFV) was calculated according to the formula: MFV = [PSV+(EDV *x*2)]/3.

It was obtained at rest by the continuous recording of 1-min period of normal breathing, then the subjects were asked to hold their breath for 30 s following a normal inspiration in order to avoid a Valsalva effect [13]. A normal BHI was considered if ≥0.69, an impaired BHI if <0.69 [19]. Subjects were preliminary instructed on how to perform BHI and they test the procedure before starting the recordings.

Three subjects were excluded because with poor insonation of the temporal bone window.

### *C*: the CIMT assessment

CIMT was evaluated as distance between the leading edge of the lumen-intima interface and the leading edge of the media adventitia interface, according to the recommendations of the Mannheim Carotid Intima-Media thickness Consensus [20]. The investigator performed not invasive measurements of CIMT using a high-resolution B-mode ultrasound (Envisor, Philips) equipped with a 7.5-to 12-MHz probe. For all subjects, CIMT was measured in the far wall of the common carotid artery proximal to the bifurcation, at the bulb and the origin of the internal carotid artery of both sides [20]. On each arterial site, mean CIMT values (as averaged across the entire distance) and maximal CIMT values were recorded using Carotid Studio, a validated real-time automatic technique for CIMT measurement [21]. These 6 combined mean and maximal CIMT far-wall measurements from left and right side were averaged.

### *D*: the diameter of the abdominal aorta

The examination took place with the subject in the supine position, using a 5 MHz phased-array probe. Aorta was visualized in the longitudinal plane and was examined from the diaphragm to the bifurcation. Aorta was then examined in the axial plane with scans perpendicular to the longitudinal plane. Aortic diameters were measured 1 cm distal to the renal arteries level.

#### Statistical analysis

A Shapiro Wilk test was used to test the null-hypotesis that the population was normally distributed.

Because of the fact that BHI, mean and max IMT resulted not normally distributed, the non-parametric Mann- Whitney's *U* test was used for these data.

For the other data, differences between two groups were analysed by a two-tailed unpaired Student’s *t*-test.

Normally distributed continuous variables (ABI and Diameter of the abdominal aorta) are expressed as mean ± SD while non-normally distributed variables (BHI, C-IMT mean and max) are presented as median and interquartile range (IQR), according to the formula: IQR = xU-xL, where xU is the Upper quartile and xL is the Lower quartile (25th -75 ^th^ percentile).

Statistical significance was inferred at p values < 0.05. All data were analysed with StatView (SaS).

## Results

Both groups of non-smokers and smokers were comparable with respect to age and gender; moreover body mass index (BMI), systolic and diastolic blood pressure (SBP and DBP) and heart rate (HR) were within normal range and similar in both groups (*p* not significantly different).

None of the subjects had significant stenosis or occlusion of the extra and intra-cranial arteries.

No differences in MCA MFV (cm/s) at rest were observed between smokers and non-smokers (*p* not significantly different).

Substantially, groups baseline differences referred only about the current smoking habit (present or absent, number of cigarettes/day and smoking duration in years) (*p* < 0.01).

The examiner spent a mean of 8 min for the total examination of the non-smoker subjects, and a mean of 8 min plus the 10 min after smoking for the smoker ones.

Table 1 depicts non-smokers and smokers demographic and clinical characteristics.

**Table 1:** Demographic and clinical characteristics of the study population setting

CHARACTERISTICS	NON-SMOKERS	SMOKERS	*p*
	(*n* = 43)	(*n* = 38)	
Age (years)	27 ± 4	26 ± 3	ns
Male/Female	21/22	18/20	ns
BMI (Kg/m2)	23 ± 2	23 ± 2	ns
Number of cigarettes/day	0 ± 0	11 ± 5	<0.01
Smoking duration (years)	0 ± 0	8 ± 2	<0.01
SBP at rest (mmHg)	115 ± 15	117 ± 5	ns
DBP at rest (mmHg)	69 ± 8	70 ± 5	ns
Heart rate at rest (bpm)	65 ± 4	66 ± 4	ns
SBP 1 min after smoking (mmHg)	nd	127 ± 4	nd
DBP 1 min after smoking (mmHg)	nd	75 ± 4	nd
Heart rate 1 min after smoking (bpm)	nd	74 ± 2	nd
SBP 10 min after smoking (mmHg)	nd	120 ± 4	nd
DBP 10 min after smoking (mmHg)	nd	70 ± 4	nd
Heart rate 10 min after smoking (bpm)	nd	68 ± 2	nd
MCA MFV at rest (cm/s)	63 ± 4	63 ± 5	ns

*BMI* body mass index; *SBP* systolic blood pressure; *DBP* diastolic blood pressure; *MCA* middle cerebral artery; *MFV* mean flow velocity; nd, not done; ns, not significantly different; data are expressed as mean ± SD

Table 2 depicts the results of the “ABCD” assessment in the study population setting.

**Table 2:** the “ABCD” assessment in the study population setting

ABCD PARAMETERS	NON-SMOKERS	SMOKERS	*p*
	(*n* = 43)	(*n* = 38)	
ABI	1.1 ± 0.05	1.1 ± 0.8	ns
BHI baseline (MCA)	1.5 (0.3)	1.2 (0.1)	<0.01
BHI 1 min after smoking (MCA)	nd	1 ± 0.2	nd
BHI 10 min after smoking (MCA)	nd	1.1 ± 0.1	nd
C-IMT (mean, mm)	0.4 (0.06)	0.65 (0.09)	<0.01
C-IMT (max, mm)	0.53 (0.01)	0.75 (0.5)	<0.01
Diameter of the abdominal aorta (mm)	15 ± 2	15 ± 1	ns

*ABI* ankle-brachial index; *BHI* breath holding index; *MCA* middle cerebral artery; *CIMT* carotid intima media thickness; nd, not done; ns, not significantly different

Normally distributed continuous variables (ABI and Diameter of the abdominal aorta) are expressed as mean ± SD while non-normally distributed variables (BHI, C-IMT mean and max) are presented as median and interquartile range (IQR)

As depicted in table 2, smokers presented higher values of CIMT (mean and maximal), and lower BHI in MCA at baseline (*p* < 0.01) with respect to non-smokers, though in the normal range.

No significant differences were found for A and D between smokers and non- smokers.

## Discussion

To our best knowledge, this is the first study aimed to create an easy to perform four-step ultrasound examination to evaluate the peripheral, the extra and the intra-cranial assessment of the arterial damage in young, otherwise healthy smokers. The ABCD consists in an easy to remember formula not only for physicians but also in an educational context. The results of this study could be the basis for detecting the same ultrasound parameters also in older populations of smokers (or with other cardiovascular risk factors) in order to create an “ultrasound score system” both for screening and follow-up.

The finding that acute cigarette smoking increased HR, SBP and DBP are consistent with previous ones [22].

Previous studies report different results about ABI assessment in smokers compared to non-smokers. In fact, in Korean adolescents (14–16 years old) there were no significant differences in ABI values between smokers compared to non-smokers [23], while in older subjects, but not elderly, current smokers had a lower mean ABI [24], showing the significant effect of smoking in the PAD development, with a tendency of lower ABI in the oldest age group.

Variations of method of ABI measurement could affect the values, while comparing Doppler method to oscillometric or auscultation ones or plethysmography, but the recent recommendation consider the Doppler method of choice, as the most reliable method with the best reproducibly, as recently reviewed [4].

BHI is proved to be effective and reproducible in the study of the cerebral hemodynamic with TCCD and has the advantage of being more rapid and well-tolerated than carbon dioxide inhalation and acetazolamide injection [25]. The approach consisting of a fixed period of apnoea of 30 s following a normal inspiration can be considered sufficient to avoid a Valsalva effect.

Our data about BHI are in accordance with previous findings [26]: in that paper Authors found the long-term impairment of vasodilator ability of cerebral vessels in young chronic smokers, also after smoking cessation and without CIMT impairment. Nevertheless, a previous study [11] found the acute effects of smoking in reducing the cerebrovascular reactivity to hypercapnia, suggesting a failure of cerebrovascular regulation that, in this case, was observed only a short time after smoking.

The lack of differences in BHI between males and females is in accordance with previous studies [27], but the velocity measurements by TCCD seems to be affected by the gender in some cases with not definitive explanations [28, 29].

The demonstration of higher CIMT values in healthy smokers than in age-sex matched non-smokers (though in the normal range) is in accordance with our previous one [8], with consistent results both in older subjects, [30, 31] and also in even younger healthy university students [32].

The debate about CIMT remains still open: although it was a widely accepted ultrasound marker of subclinical atherosclerosis, recently it has been shown that the traditional cardiovascular risk factors, and so also smoking, can explain only a small proportion of its variance [33]. Moreover, there are no international guidelines on how precisely this measurement should be applied as a research tool, as recently reviewed in [34], and technical issues associated with CIMT measurements are still open.

As regards the evaluation of *D*, this is an important point. Atherosclerosis and AAA share risk factors, such as smoking, obviously, and also hypertension, and hypercholesterolemia [15], but many patients with advanced atherosclerosis do not develop AAA, whereas many patients without evidence of atherosclerosis present AAA. Cigarette smoking is surely the strongest risk factor for AAA. The strength of association between smoking and AAA is 2.5 times greater than the association between smoking and CAD, and 3.5 times greater than the association between smoking and cerebrovascular disease [35].

This is the first study that take together into account the indexes considered in the “ABCD” assessment, in particular in young healthy subjects.

The attempt to consider different parameters to assess the risk for smokers has been done in previous reports: for example, the Multi-Ethnic Study of Atherosclerosis (MESA) [36] considered coronary artery calcification, CIMT and ABI, in conjunction with circulating markers of inflammation in older patients.

It is reasonable that smoke affects MFV and, as reported in previous studies [37–39] also the vasomotor activity. It is possible that a subtle increase in MFV reflects a more subtle and generalized arterial narrowing caused by atherosclerosis. MFV increase could substantially represent a marker of intracranial atherosclerosis. In fact, as previously demonstrated [40], the highest MFV was not associated with the side of the stroke, and this observation supports the hypothesis that increased MFV reflects a generalized process providing evidence that there is a strong independent association between MCA MFV and the risk of stroke (in particular the ischemic one).

The association between altered cerebrovascular reactivity and the degree of coronary atherosclerosis has been found [41] and it was confirmed also in patients without carotid stenosis [14], so the link between CAD and cerebrovascular functional impairment has been supported.

This study has several limitations. First of all, Authors have not yet provided a follow up assessment for the subjects (it could be useful to detect any damage progression within 1 or more years from the baseline results, as assessed for what concerns CIMT in previous report [32]). Moreover, it has not been tested the effects of acute smoking in non-smokers, as other Authors have analysed in order to speculate about the acute smoking-induce hemodynamic alterations in the common carotid artery [25], this choice for ethical reasons. Nevertheless, it is a matter of fact that cigarette smoking has a specific fibrogenic effect which causes intimal thickening, as previously demonstrated [42, 43].

We have discussed about this topic in a recent study [8].

In that study we aimed to assess whether cigarette smoking-induced oxidative stress affects lipoprotein associated phospholipase A2 (Lp-PLA2) expression in peripheral blood mononuclear cells (PBMC) and hence Lp-PLA2 and lysoPC plasma concentrations, as well as the relationship between lysoPC and CIMT in healthy smokers. The correlation between lysoPC and CIMT together with the finding that lysoPC up-regulates proteoglycan synthesis suggests that lysoPC may be a link between smoking and intimal thickening.

On the basis of these results, it is reasonable to consider that smoking has a “physical” effect on CIMT, it is not only a physiologic adaptation.

It is to mention that BHI is considered the most practical, inexpensive, best-tolerated and easy to perform alternative to the previous methods, as previously explained [13]. Nevertheless, to reach this goal, almost all studies report that subjects, even young, must be instructed before the recordings [13, 44]. This process allows good compliance and reliable results. Also in the present study, subjects were preliminary instructed on how to perform BHI and they test the procedure before starting the recordings.

Even so, older subjects may be unable to complete the test, as previously described [45].

Further studies are needed to test the same “ABCD” also in patients with different cardiovascular risk factors and ages, nevertheless, our results seems to underline the importance of the assessment of *B* and *C*, that, though in the normal range, present significant differences between young healthy smokers and non-smokers. These results could drive the screening between smokers in age-related manner.

## Conclusions

The “**ABCD**” examination could represent a valid and quick (8 min) method to detect and then monitor smokers’ vascular damage, and could be the systematic approach to be considered also for older smokers. Finally, this approach should be contemplated in the wider context of possible not-invasive and practical screening and follow-up modalities. This would be designed to implement preventive strategies and tools aimed at discouraging tobacco addiction and monitoring cardiovascular risk patients.

Accurate assessment of cardiovascular risk is an essential goal to be achieved, and many attempts to collect reliable vascular biomarkers have been made, as recently reviewed [46], for primary and secondary prevention.

Although the “ABCD” examination is now far to be used as a screening tool, it could be considered a further attempt in this area.

## Abbreviations

AAA, abdominal aortic aneurysm; ABI, ankle-brachial index; BHI, breath-holding index; BMI, body mass index; CAD, coronary artery disease; CIMT, carotid intima media thickness; DBP, diastolic blood pressure; HR, heart rate; MCA, middle cerebral artery; MFV, mean flow velocity; PAD, peripheral artery disease; SBP, systolic blood pressure; TCCD, trans-cranial colour Doppler
